# Supplementary material for: Defining the condensate landscape of fusion oncoproteins
Source: Nat Commun. 2023 Sep 28;14:6008. doi: 10.1038/s41467-023-41655-2 (PMC10539325; doi:10.1038/s41467-023-41655-2)
Supplement: Supplementary file 1 — Supplementary Information file [file 41467_2023_41655_MOESM1_ESM.pdf]

Supplementary Figures and Legends

Supplementary Fig. 1

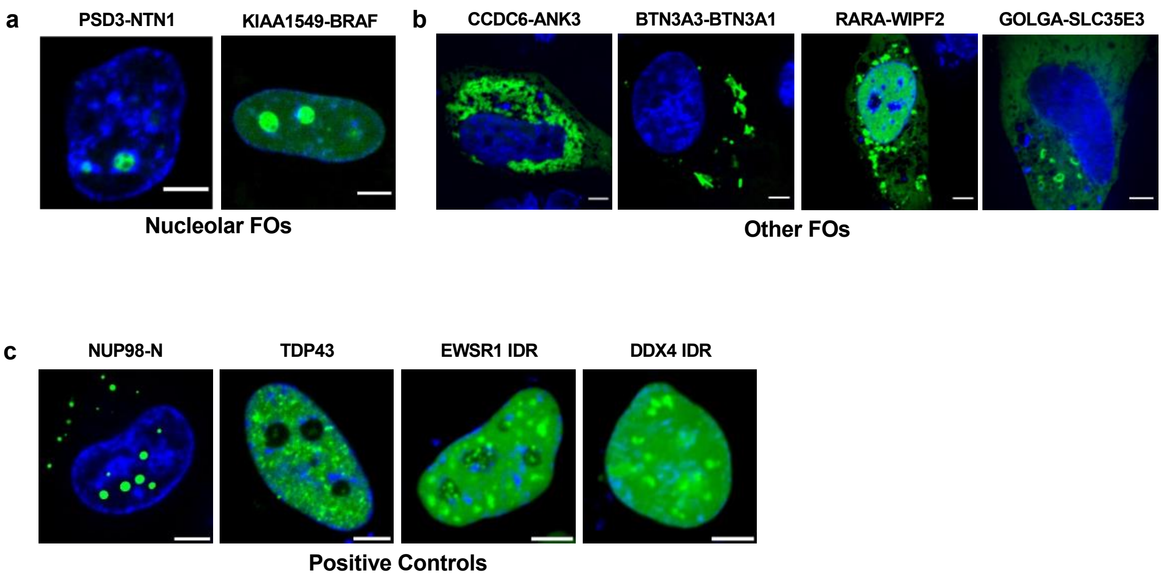

Supplementary Figure 1. Representative live cell images of mEGFP-tagged FOs classified as "Nucleolar" or "Other" and condensate-forming positive controls. Related to Figure 2. (A, B) Representative confocal microscopy images of live HeLa cells expressing mEGFP-tagged FOs classified as Nucleolar (A) or Other (B), based upon two biological replicates. (C) Representative confocal microscopy images of live HeLa cells expressing mEGFP-tagged NUP98-N or mCherry-tagged TDP43, EWSR1 IDR, or DDX4 IDR, based upon two biological replicates. FO signal (green) is overlayed with the DNA signal (Hoechst dye, blue). All scale bars are 5 µm.

Supplementary Fig. 2

a

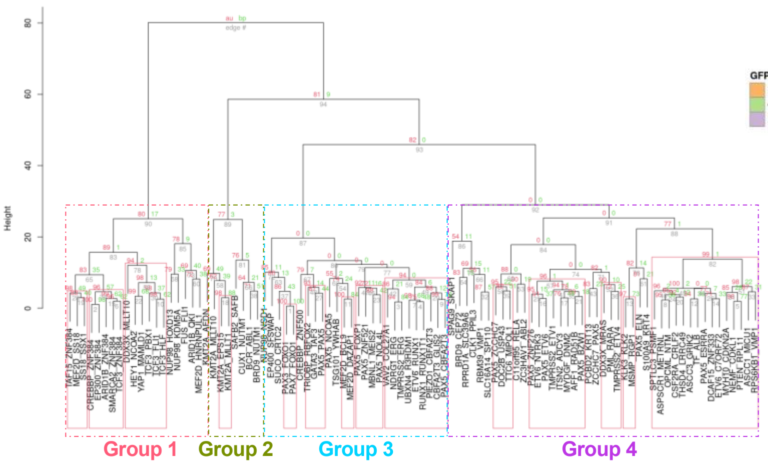

b

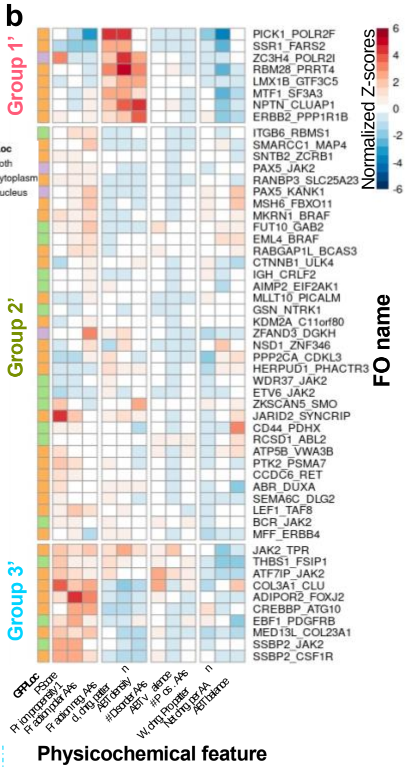

c

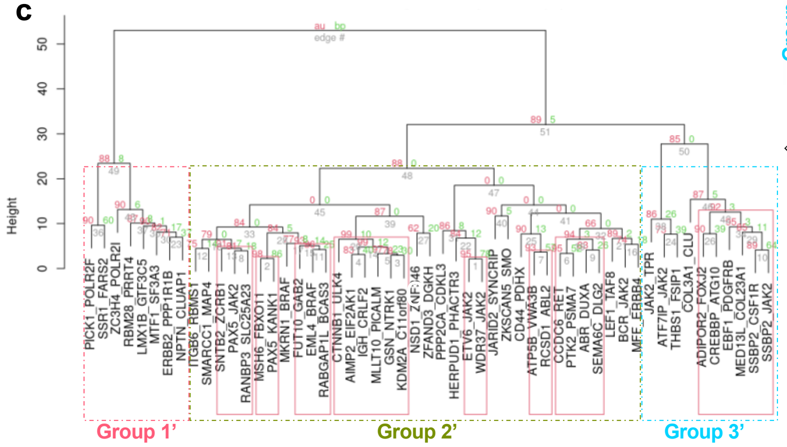

d

Average sequence identity (SI_{av}) values for puncta(-) FOs within a group		
Group 1'	Group 2'	Group 3'
7.9 ± 0.4 %	9.7 ± 0.3 %	14.4 ± 2.0 %

e

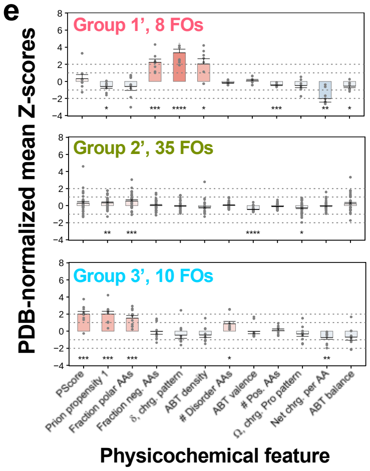

f

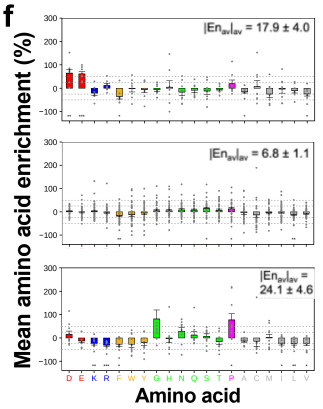

Supplementary Figure 2. Significance of clustering of puncta(+) and puncta(-) Expressed FOs and physicochemical features of the puncta(-) Expressed FOs.

(A) Assessment of uncertainty in hierarchical clustering of physicochemical features for the 96 puncta(+) FOs. Approximately unbiased (AU) and Bootstrap Probability p-values (%) are indicated in red and green color, respectively. Clusters of FOs with AU p-values ≥ 90% are highlighted by red rectangles and are considered to be statistically significant. The dashed rectangles indicate the FOs in the feature groups (Groups 1-4) shown in Fig. 3D.

(B) Two-dimensional (2D) hierarchical clustering of the puncta(-) FOs on the basis of the 12 most discriminatory physicochemical features. FO names are reported on the vertical axis. Values of features are reported on the horizontal axis. The first column (left) represents localization of the diffuse mEGFP fluorescence. FOs cluster into three groups (Groups 1’-3’). See Supplementary Dataset 5 for information on the physicochemical features used.

(C) Assessment of uncertainty in hierarchical clustering of the 53 puncta(-) FOs. Clusters of FOs with AU p-values ≥ 90% are highlighted by red rectangles and are statistically significant. The dashed rectangles indicate the FOs in the feature groups (Group 1’-3’) shown in (B).

(D) Average sequence identity +/- standard error for pairwise comparison of all FOs within each of the groups in (B).

(E) Quantification of the mean enrichment or depletion of the 12 physicochemical features for puncta(-) FO Groups 1’-3’. Values are reported as mean Z-scores +/- standard error and normalized to the averages of the human PDB sequences. Z-score values of the puncta(-) FOs for each feature are shown in solid gray circles along the y-axis for Groups 1’-3’. Average values of the absolute mean Z-scores +/- standard error are reported in the top right of each plot.

(F) Average values of the absolute mean amino acid enrichment or depletion +/- standard error for sequences of puncta(-) FOs in feature Groups 1’-3’. The amino acid enrichment values of the puncta(-) FOs are shown in solid gray circles along the y-axis. The mean of the absolute

average enrichments +/- standard error are reported in the top right of each plot. In both (E) and (F) significance was calculated using two sided t-test with respect to the human sequences in the averages of the human PDB sequences and no adjustment were made for multiple comparisons (*, $p < 0.05$; **, $p < 0.01$; ***, $p < 0.001$; ****, $p < 0.0001$). Source data are provided as a Source Data File.

Supplementary Fig. 3

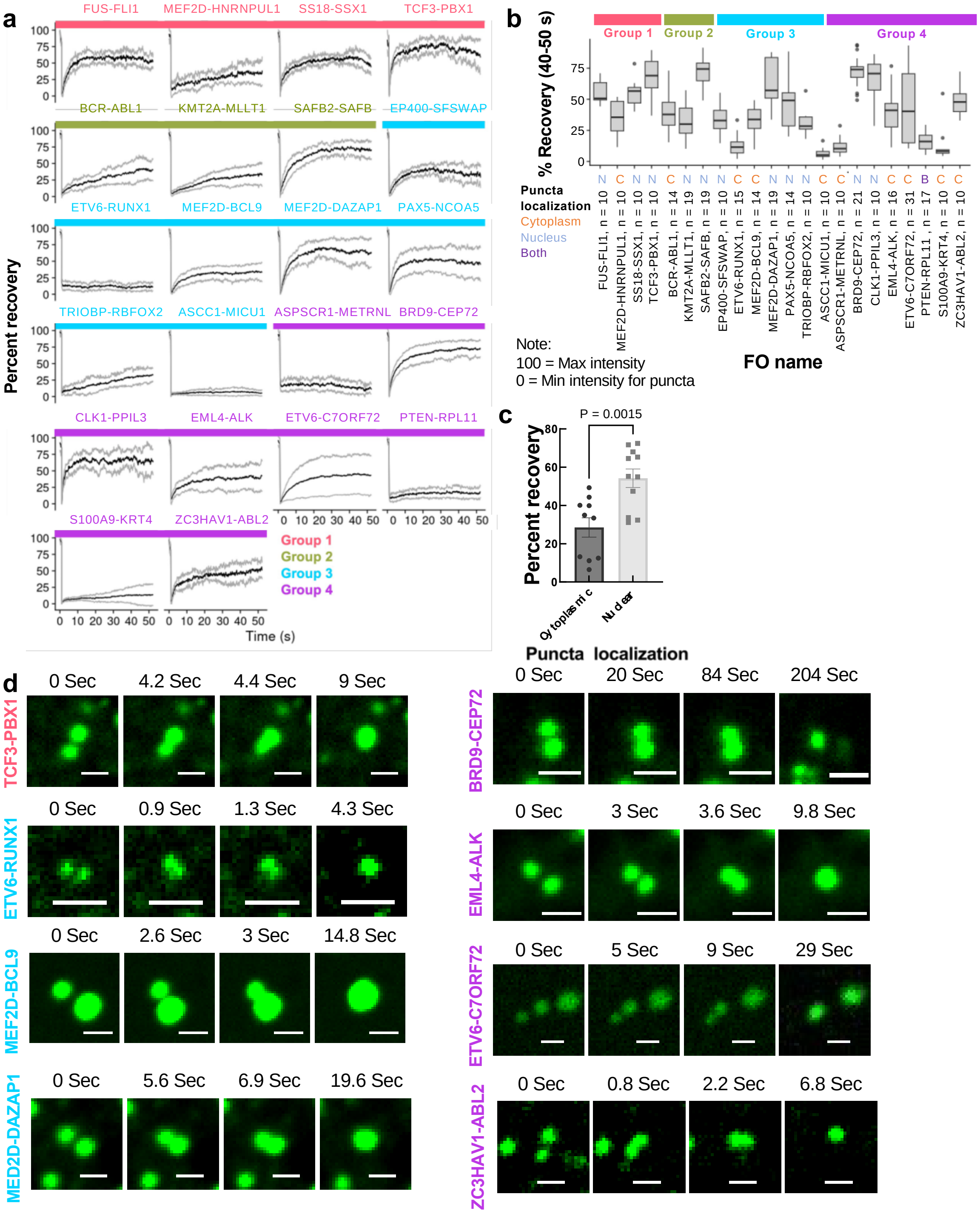

Supplementary Figure 3. Probing the physical properties of puncta(+) FO condensates. (A) Average FRAP traces for 22 puncta(+) FOs. Data is plotted as mean (black trace) +/- standard error. At least 20 puncta were analyzed for each FO. Colored bars reflect the physicochemical feature group of each FO (Groups 1-4, as defined in Fig. 3D and indicated at the bottom).

(B) Quantification of the average normalized FRAP percent recovery of mEGFP fluorescence for FOs between 40-50 seconds after bleaching. Box plots report the median values as a center line, with boxes extending to the first and third quartiles. Lines extend from the edges of each box to include all values within the interquartile range, with outliers outside of that range represented as points above or below the box and whisker plot. Colored bars reflect the physicochemical feature group of each FO. Localization is indicated by N (nucleus), C (cytoplasm), or B (both).

(C) Quantification of the average normalized FRAP percent recovery of mEGFP fluorescence for FOs between 40-50 seconds after bleaching. Data is compared for FOs localized to either the cytoplasm (n=11) or nucleus (n=10). Bar plots show mean percent recovery +/- standard error, and values of individual FOs are shown as points. Significance was assessed using the two-sided t-test and no adjustment were made for multiple comparisons.

(D) Still images of multiple time-points taken from a time-lapse confocal fluorescence microscopy video of fusion events in a HeLa cells expressing the respective mEGFP-tagged FOs. All scale bars are 1 μm. Source data are provided as a Source Data File.

Supplementary Fig. 4

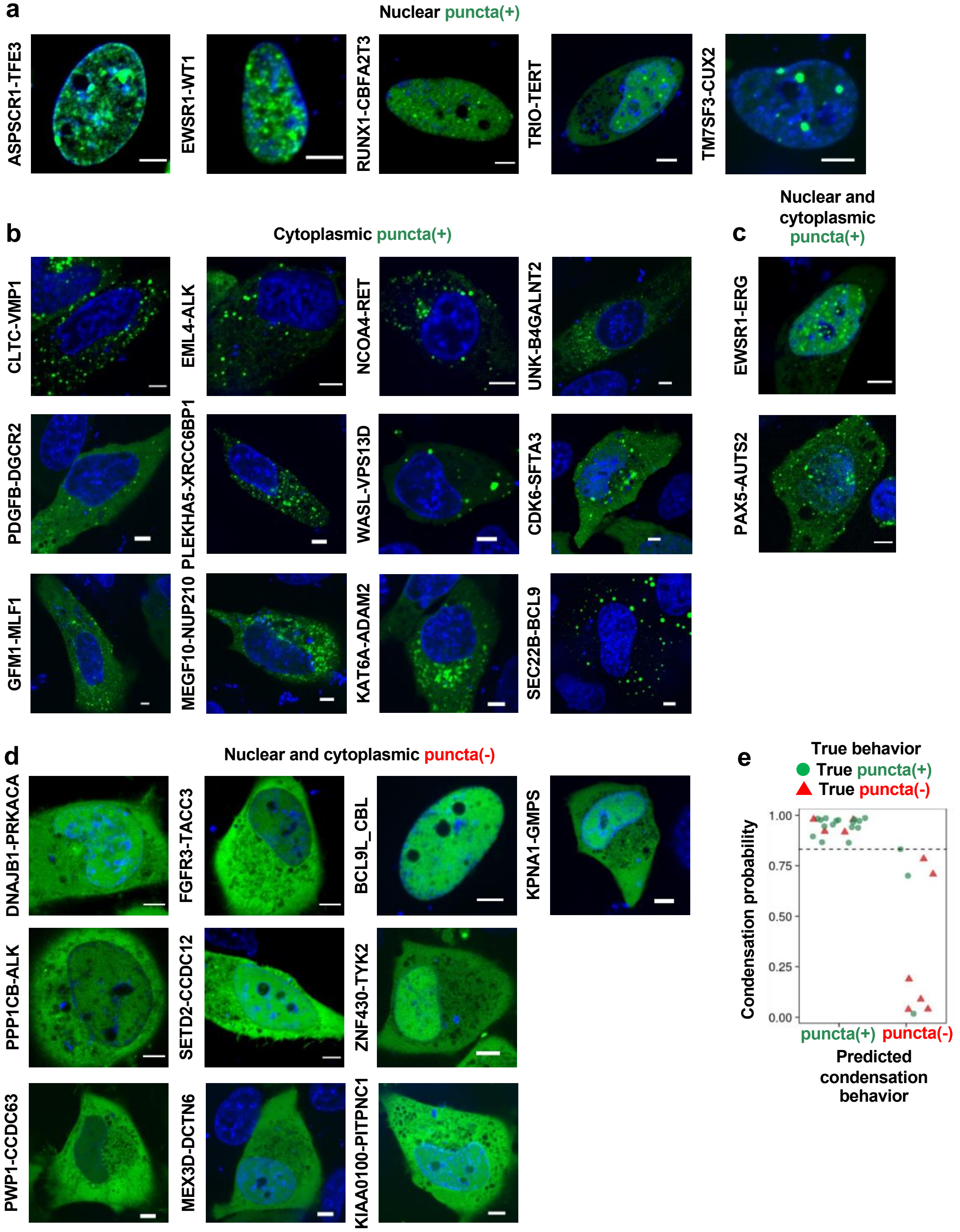

65

66

Supplementary Figure 4. Verification of FO-Puncta ML model performance. Related to Figure 5.

(A-D) Representative confocal microscopy images of live HeLa cells expressing the 29 mEGFP-tagged Verification FOs. Several of these FOs formed puncta in the nucleus (A), cytoplasm (B), or both the nucleus and cytoplasm (C), while other exhibited diffuse localization in the nucleus and cytoplasm (D), based upon two biological replicates. In all images, the FO signal (green) is overlayed with the DNA signal (Hoechst dye, blue). All scale bars are 5 μm.

(E) Results of prediction of condensate formation behavior [x-axis, puncta(+) or puncta(-)] using the FO-Puncta ML model for the 29 Verification FOs. Predicted condensate formation probability from the FO-Puncta ML model is along the y-axis. The true puncta(+) FOs are depicted with green circles, while the true puncta(-) FOs are depicted with red triangles. The horizontal dotted line indicates the condensate formation probability value of 0.83, which was used as the threshold for predicting puncta(+) behavior. This value was determined by assessing the maximum F1 score for the FO-Puncta ML model tested with different threshold values, where F1 is the harmonic mean of the precision and recall based on the optimal performance of the model with the 29 Verification FOs. Source data are provided as a Source Data File.

Supplementary Fig. 5

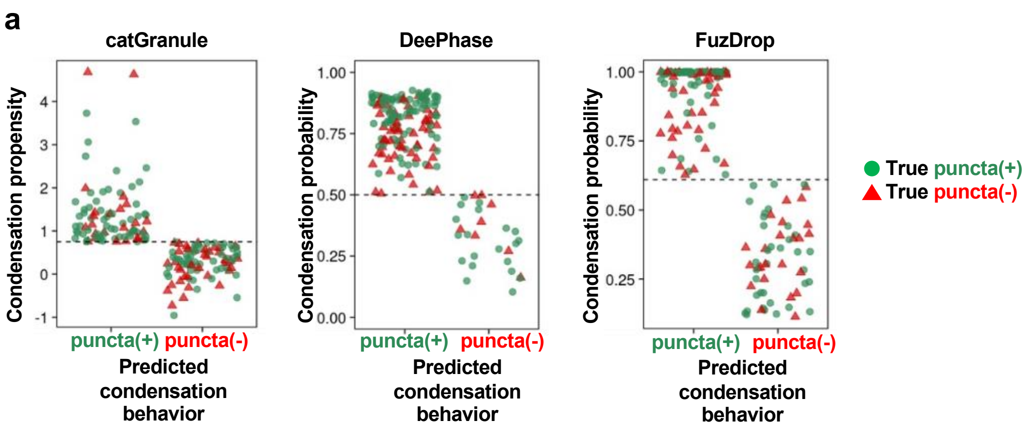

Supplementary Figure 5. Comparison of the FO-Puncta ML model with other phase separation prediction models. Related to Figure 5.

(A) Results of the predicted condensation behavior (x-axis) from three previously developed phase separation predictors (catGranule, DeePhase, and FuzDrop) applied to our combined Training and Verification FOs. The true (experimentally determined) puncta(+) FOs are depicted with green circles, while true puncta(-) FOs are depicted with red triangles. Predicted condensate propensities/probabilities from the different predictors are shown along the y-axis. Source data are provided as a Source Data File.

Supplementary Fig. 6

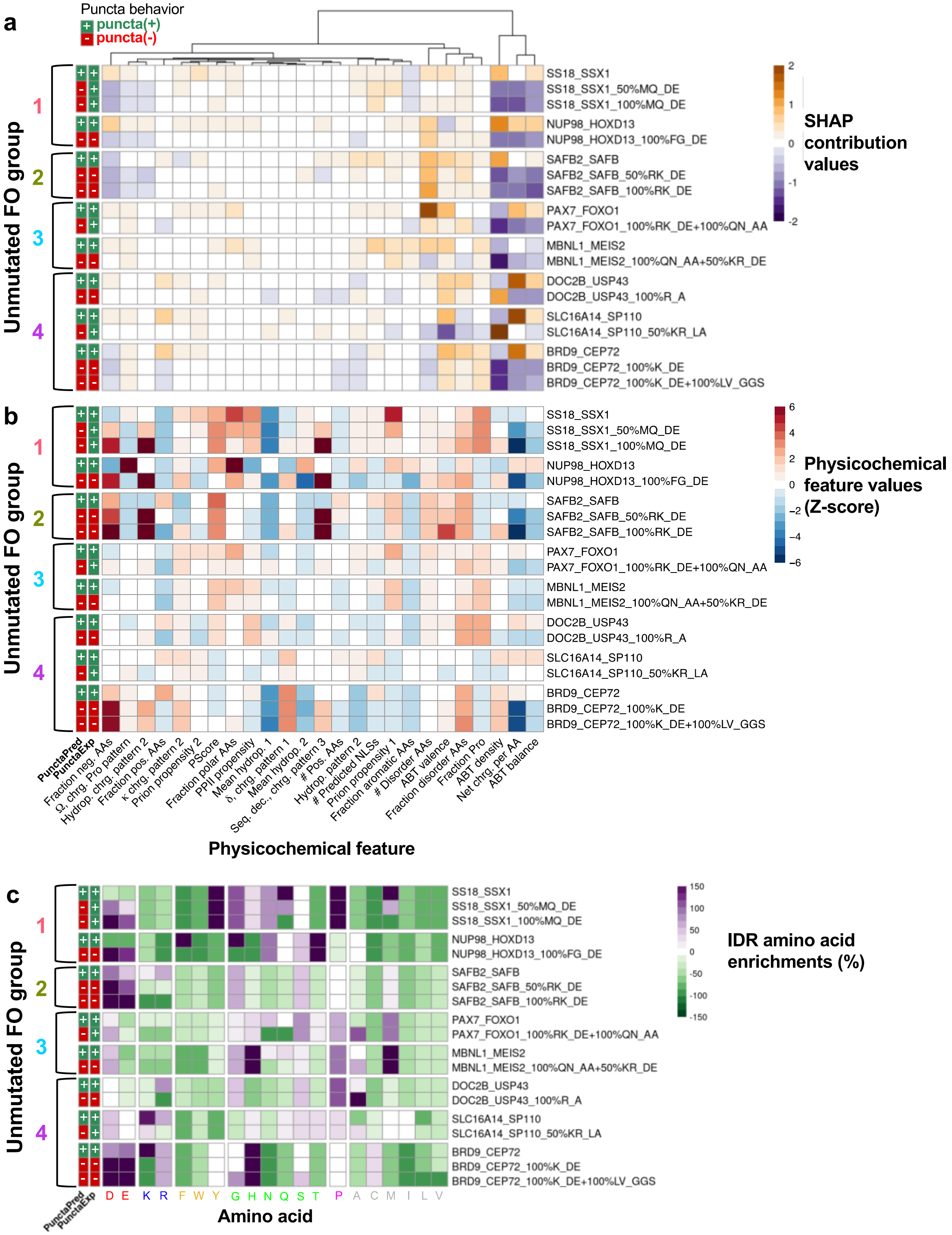

Supplementary Figure 6. Related to Figure 6. Mutagenesis of puncta(+) Expressed FOs from Groups 1-4. Related to Figure 6.

(A-C) SHapley Additive exPlanations (SHAP) contribution values (A), 25 physicochemical feature values (B), and intrinsically disordered region (IDR) amino acid enrichments (C) for unmutated and mutated FOs presented as clustered heatmaps. The types of introduced mutations are given to the right of the FO names The first column provides the FO-Puncta ML model prediction (PunctaPred), and the second column provides the experimentally determined condensation behavior (PunctaExp). The group (Groups 1-4) from which each original FO is derived is indicated on the left. See Supplementary Dataset 7 for additional information on mutation strategy and Supplementary Dataset 5 for additional information on the physicochemical features used in these analyses. Source data are provided as a Source Data File.

Supplementary Fig. 7

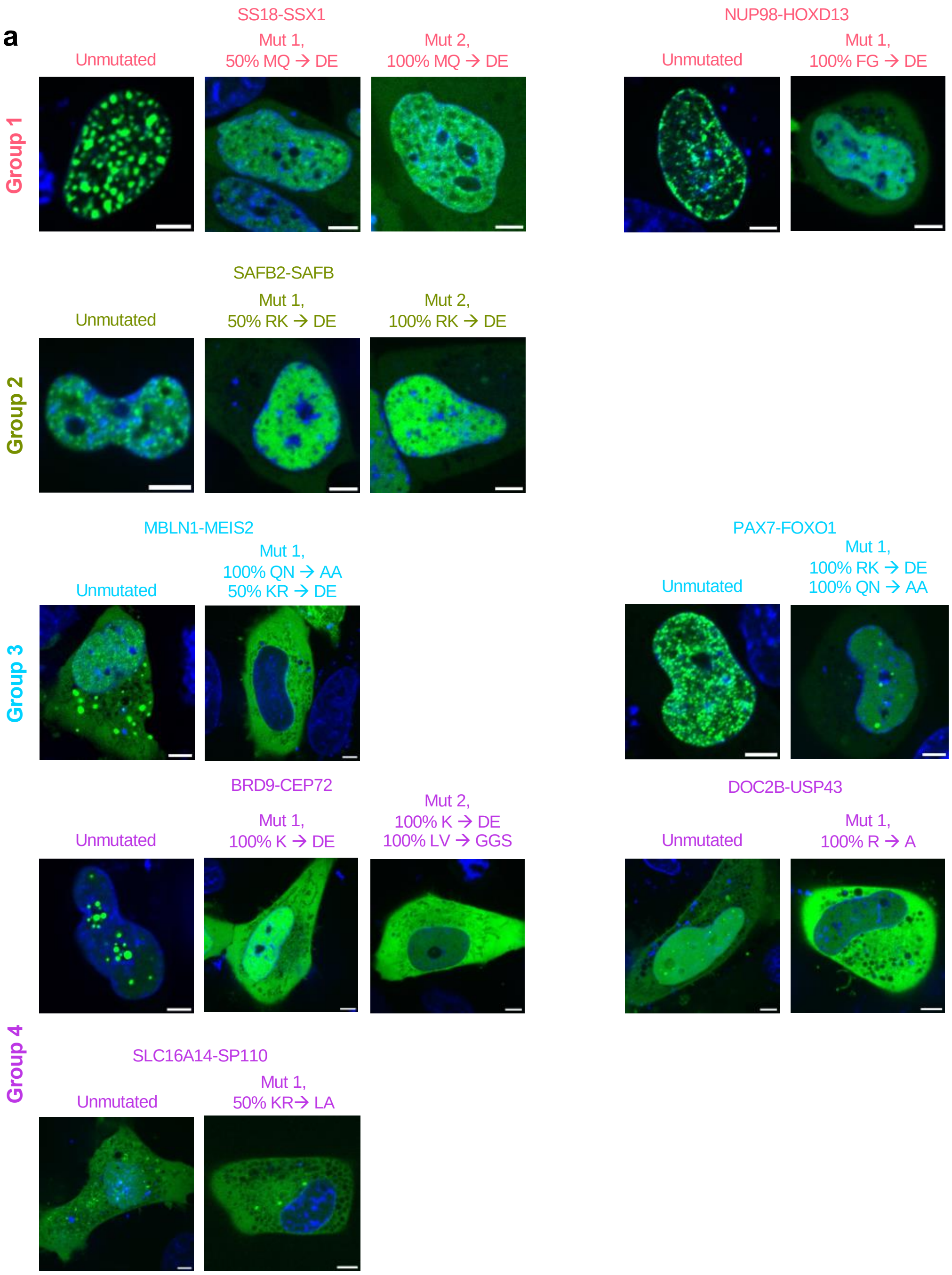

Supplementary Figure 7. Representative live cell images of mEGFP-tagged unmutated and mutated FOs. Related to Figure 6.

(A) Representative confocal microscopy images of live HeLa cells expressing mEGFP-tagged unmutated or mutated FOs, based upon two biological replicates. In all images, the FO signal (green) is overlayed with the DNA signal (Hoechst dye, blue). All scale bars are 5 μm.

Supplementary Fig. 8

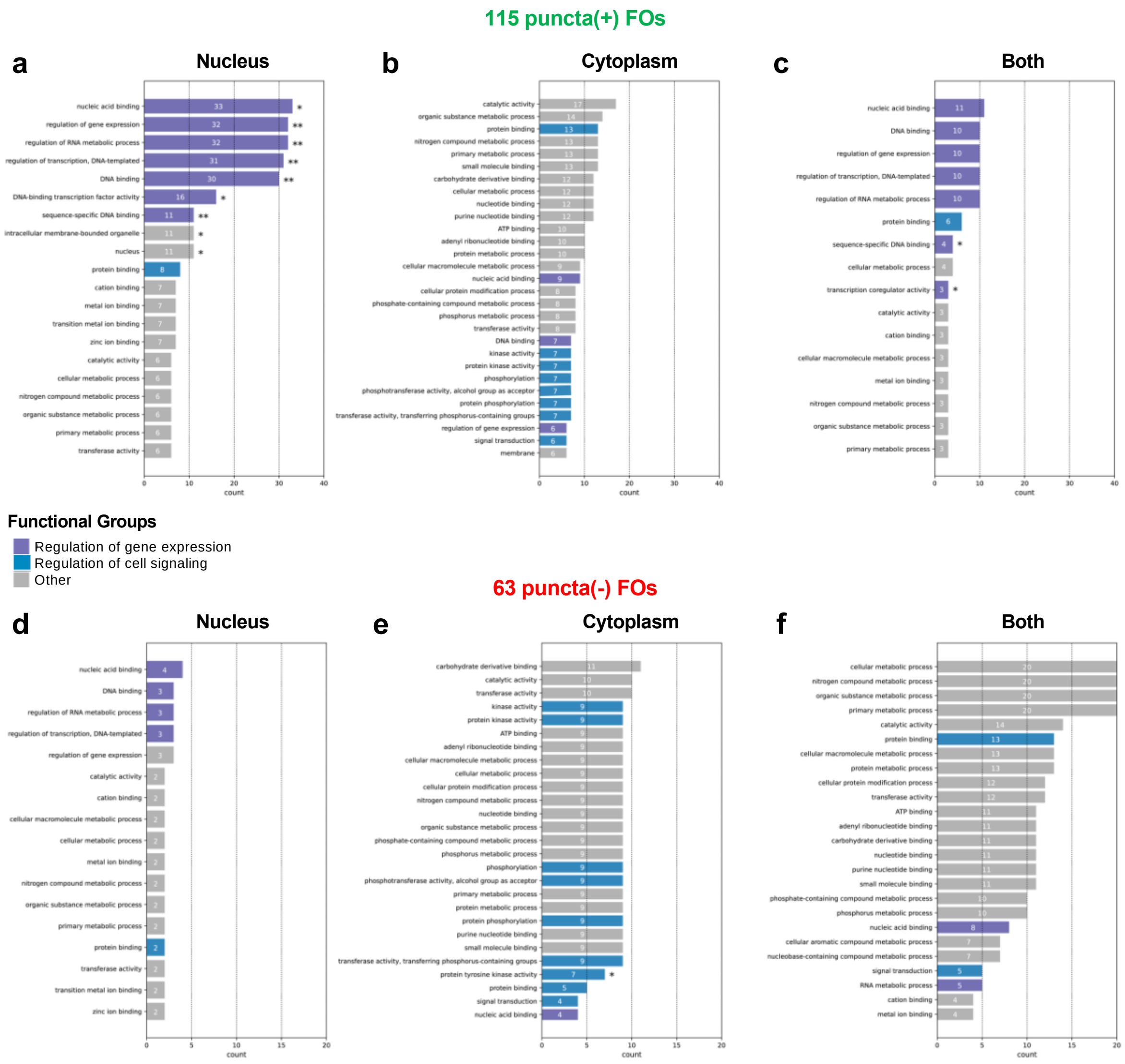

Supplementary Figure 8. Functional analysis of puncta(+) and puncta(-) Training and Verification FOs using InterPro domain GO slim annotation, including all parent terms. Related to Figure 7.

(A-C) GO "slim" terms are shown for the 115 puncta(+) Training and Verification FOs localized to the nucleus (A), cytoplasm (B), and both compartments (C).

(D-F) GO "slim" terms are shown for the 63 puncta(-) Training and Verification FOs localized to nucleus (D), cytoplasm (E), and both compartments (F). The colors of the bars represent the three major functional groups: regulation of gene expression (including transcription, chromatin, and RNA binding-related GO terms; purple), regulation of cell signaling (including protein kinase, protein binding, and cell signaling-related GO terms; blue) and other functions (grey). The numbers in each bar indicate the number of unique FOs with the noted functional term, and asterisks indicate statistically significant over-representation based on p-value estimates from 100,000-fold one-sided resampling with replacement using identically-sized protein sets (*, $p < 0.05$; **, $p < 0.01$; ***, $p < 0.001$). Source data are provided as a Source Data File.

Supplementary Fig. 9

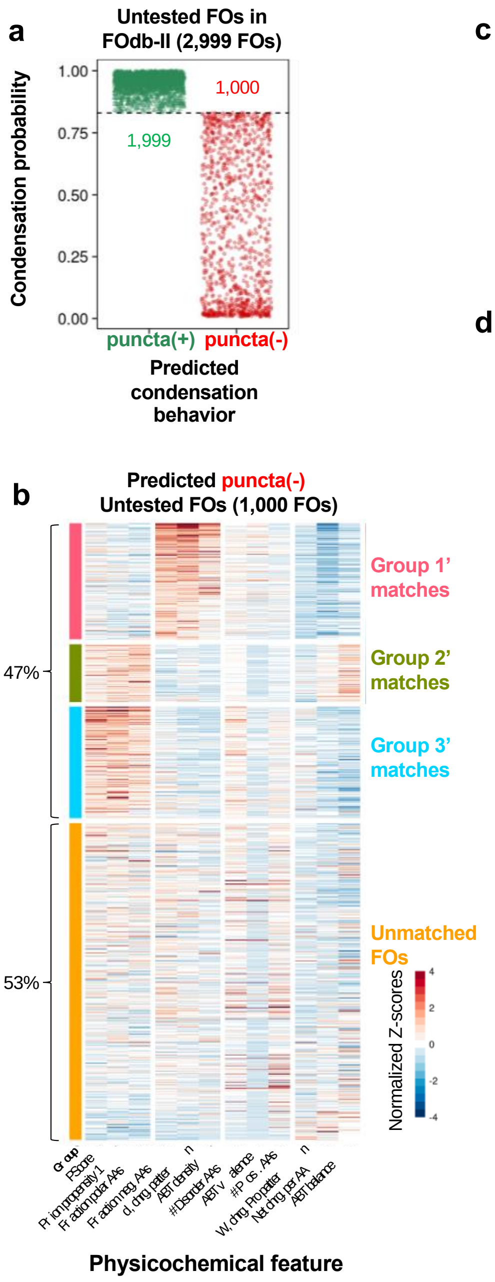

c

Average correlation coefficients ($R_{Pearson}$ mean ± SE) for pairwise matches of average feature values for puncta(-) Training FOs (Groups 1’-3’) and feature values of individual predicted puncta(-) Untested FOs			
Group 1’ matches	Group 2’ matches	Group 3’ matches	Groups 1’-3’ vs Unmatched
0.7 ± 0.0	0.7 ± 0.0	0.7 ± 0.0	0.3 ± 0.0

d

Average sequence identity (SI_{av}) of the matching feature groups between puncta(-) Training FOs and predicted puncta(-) Untested FOs	
Group matches	SI_{av}
Group 1’ (193 FOs)	7.2 ± 0.0 %
Group 2’ (96 FOs)	7.2 ± 0.0 %
Group 3’ (185 FOs)	7.8 ± 0.1 %

Supplementary Figure 9. Physicochemical features for predicted puncta(-) FOs. Related to Figure 8.

(A) Results of predicted condensation behavior using the FO-Puncta ML model for the 2,999 Untested FOs. The predicted puncta(+) FOs are depicted in green, while the predicted puncta(-) FOs are depicted in red. The FO-puncta ML model threshold for puncta(+) classification is a probability value greater than or equal to 0.83, and puncta(-) classification is a probability value less than 0.83; this threshold is indicated by the dashed horizontal line.

(B) Results of comparing the values of 12 physicochemical features (as used for the Training FOs) for each predicted puncta(-) FO in the Untested FO set to the average feature values of the puncta(-) groups (Groups 1'-3') for the Training FOs. The Untested FOs were matched to the feature groups with which they had the greatest and most significant ($p \leq 0.05$) pairwise positive correlation and data is presented as a clustered heatmap. 526 FOs (53%) did not match to any of the three groups and were placed in a separate group (Unmatched FOs, orange). See Supplementary Dataset 5 for additional information on the physicochemical features used in these analyses.

(C) The average Pearson correlation coefficients for the feature group matches displayed in (B). Data is reported as $R_{Pearson}$ mean +/- standard error.

(D) Pairwise amino acid sequence identities reported as average percent identity +/- standard error between FOs in the matched groups (Training FOs *versus* Untested FOs in Groups 1'-3'). Source data are provided as a Source Data File.

Supplementary Fig. 10

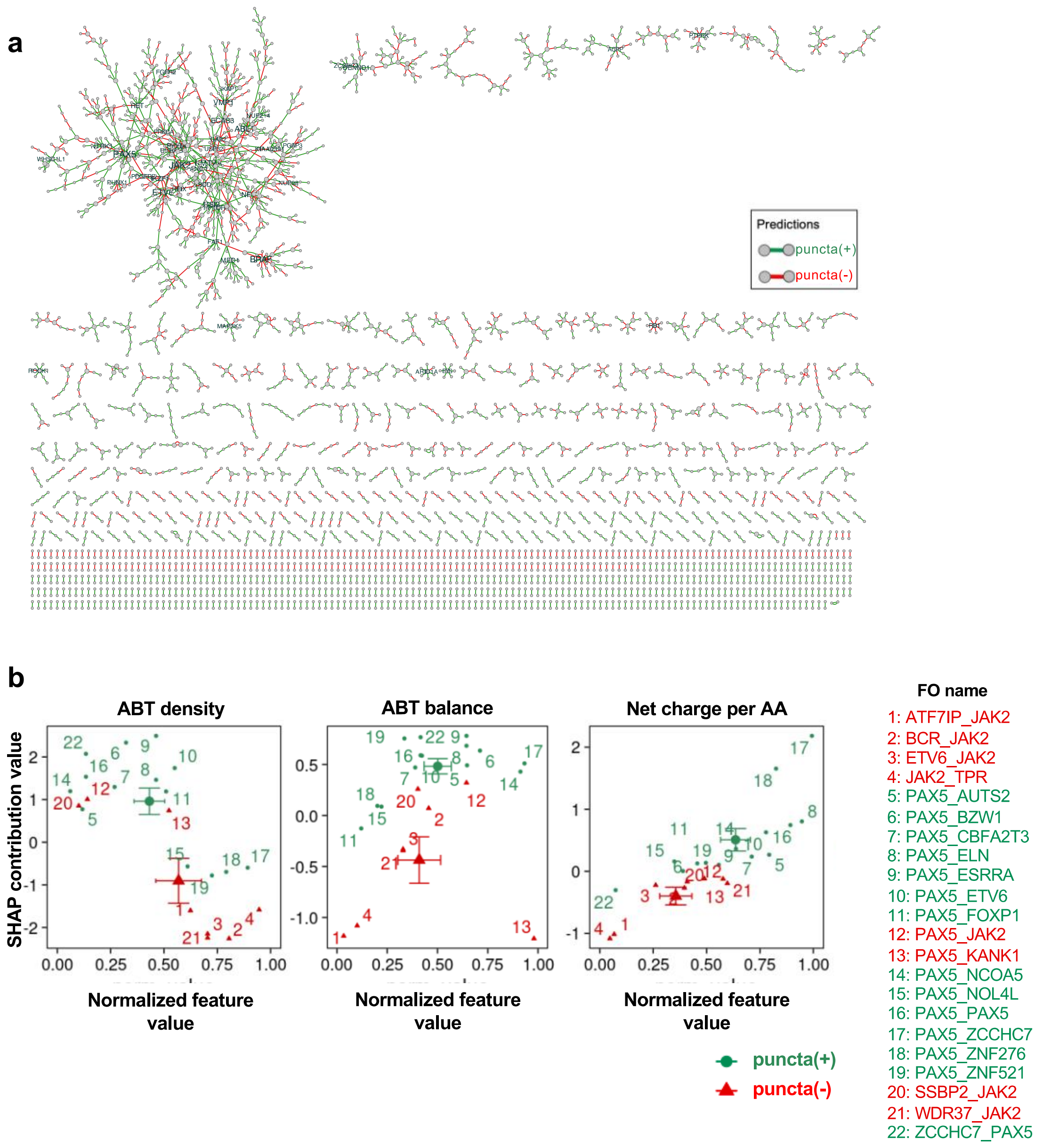

Supplementary Figure 10. Untested FO network analysis and SHAP analysis. Related to Figure 9.

(A) Cytoscape network analysis of all FO parents (nodes) from the Untested FO set (2,999 total). Edges indicate a fusion event. Green edges reflect predicted puncta(+) and red edges predicted puncta(-) FOs.

(B) For all JAK2 and PAX5 FOs (n=22) in the Training and Verification FOs, the normalized feature values and SHAP contribution values of three physicochemical features were analyzed: ABT density (left), ABT balance (middle), and Net charge per AA. Puncta(+) FOs are indicated by green circles and puncta(-) FOs by red triangles. In each plot the mean values +/- standard error for the normalized feature values and SHAP contribution value are shown by green circles and red triangles, respectively for the puncta(+) and puncta(-) FOs along with the errors bars. Source data are provided as a Source Data File.

Supplementary Fig. 11

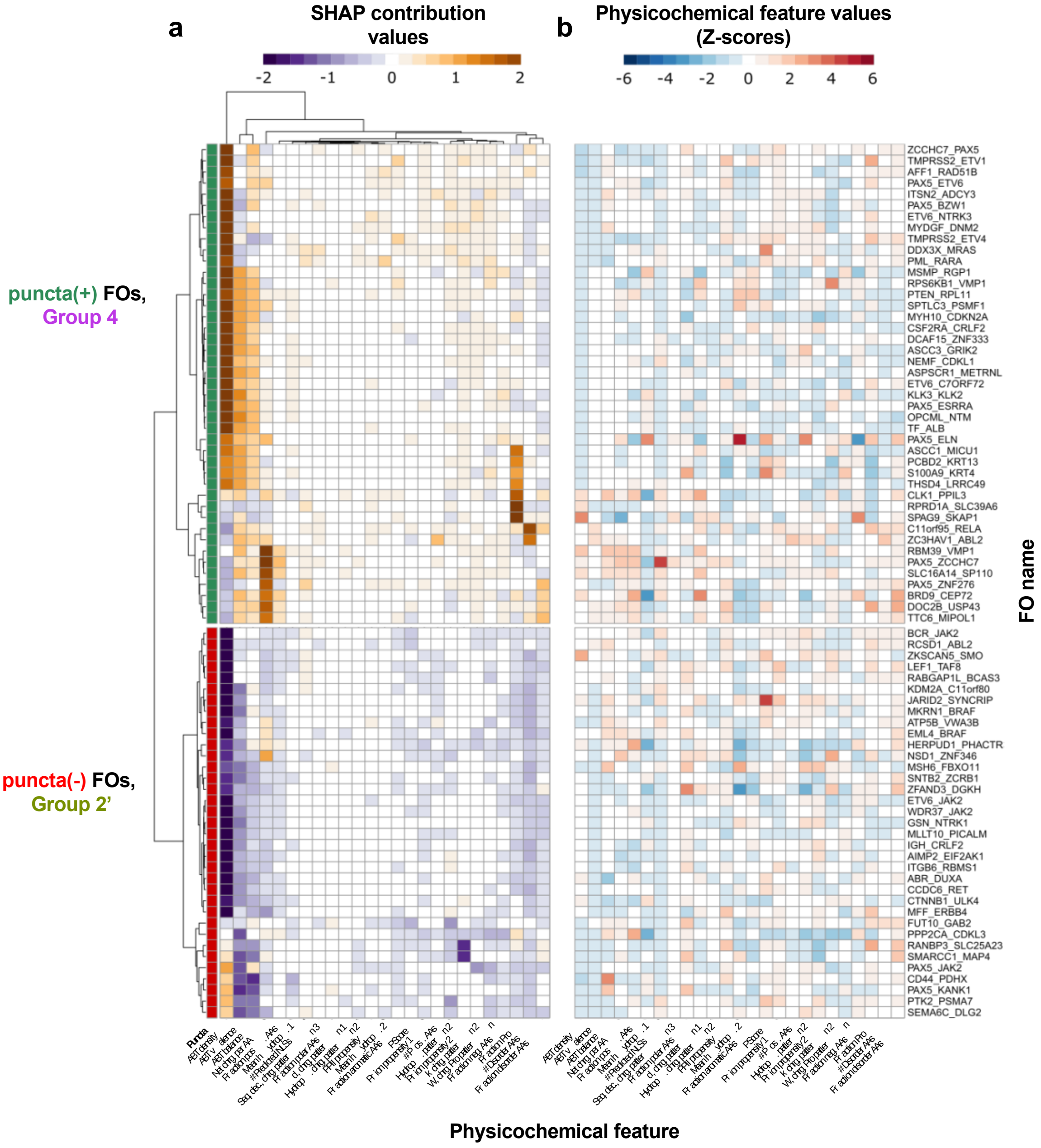

Supplementary Figure 11. Comparison of SHAP contribution and physicochemical feature values for FOs in puncta(+) Group 4 and puncta(-) Group 2'.

(A-B) SHapley Additive exPlanations (SHAP) contribution values (A) and the 25 physicochemical feature values reported as Z-scores (B) for puncta(+) Group 4 FOs and

puncta(-) Group 2’ FOs presented as clustered heatmaps. Note that unbiased clustering on the basis of SHAP contribution values resulted in full separation of the Group 4 and Group 2’ FOs. See Supplementary Dataset 5 for additional information on these and other physicochemical features used in these analyses. Source data are provided as a Source Data File.
