# Supplementary material for: Defining the condensate landscape of fusion oncoproteins
Source: Nat Commun. 2023 Sep 28;14:6008. doi: 10.1038/s41467-023-41655-2 (PMC10539325; doi:10.1038/s41467-023-41655-2)
Supplement: Supplementary file 3 — Description of additional supplementary files [file 41467_2023_41655_MOESM3_ESM.pdf]

Description of Additional Supplementary Files Document

Supplemental Dataset Legends:

Supplementary Dataset 1. The fusion oncoprotein database (FOdb).The FOdb lists fusion oncoprotein names as well as their sources and amino acid sequences. When available, FOdb includes information on numbers of patients and the cancers in which FOs were observed. See Supplementary Dataset 3 for cancer abbreviation definitions.

Supplementary Dataset 2. The FOdb-II.
The FOdb-II lists FOs for which at least 1 patient was identified. This table includes the amino acid sequences and condensate formation probability values generated using the FO-Puncta ML model and physicochemical feature group assignments, when available. Additionally, it provides information on numbers of patients and the cancers in which FOs were observed. See Supplementary Dataset 3 for cancer abbreviation definitions. For each entry, amino acid enrichments, as well as the values of the physicochemical features used to generate FO-Puncta ML Model predictions and feature group assignments are listed. Definitions of physicochemical features are found in Supplementary Dataset 5.

Supplementary Dataset 3. Cancer type abbreviations and FO set definitions.
Definitions of cancer type abbreviations and FO sets used in this study. This dataset provides cancer type abbreviations found in various Supplementary Datasets and figures in the manuscript. All referenced subsets of FOs are also defined here.

Supplementary Dataset 4. Expressed and Verification FO sets.
The Expressed and Verification FO sets were experimentally tested for puncta formation in HeLa cells. In addition to providing information on puncta occurrence and sub-cellular location, this table details biological functions associated with conserved domains identified within FO amino acid sequences and, if available, whether driver status has been demonstrated in the

literature, whether or not an oncogenic mechanism of action has been determined, relevant citations, and values of parameters generated by the SAK sequence analysis tool.

Supplementary Dataset 5. Physicochemical features used in analyses of FOs.
This table describes the 39 physicochemical features computed for the FOs examined in this study. In addition to the definition and source of the feature, the table indicates whether a feature was used in the development of the FO-Puncta ML Model and/or physicochemical feature group assignments.

Supplementary Dataset 6. Performance metrics for the FO-Puncta ML Model and other phase separation predictors with FOs examined in this study. Performance metrics for the FO-Puncta ML Model obtained independently with the Training (149 FOs) and Verification (29 FOs) FOs, and condensate formation prediction results using three previously developed phase separation predictors, catGranule, DeePhase and FuzDrop, for the combined Training and Verification FOs.

Supplementary Dataset 7. Mutational analysis of puncta(+) FOs. This table describes the rationale for mutation of selected puncta(+) FOs to modulate their condensation behavior by identifying the disordered regions (IDRs) and amino-acid enrichments within those IDRs, physicochemical features of the FOs, SHAP contributions of the features from the FO-Puncta ML model along with the predicted puncta formation probability and percentage of puncta positivity validated experimentally.

Supplementary Dataset 8. Conserved Domain (CD) analysis of the Training and Verification FOs. This table provides the conserved domains that were identified from the NCBI Conserved Domain Database (CDD) for the experimentally tested puncta(+) and puncta(-) FOs. When sufficient information was available, a functional term that describes the domain's predicted biological function was extracted from the Conserved Domain description. Functional terms could not be assigned for identified Conserved Domains there were undefined or of unknown function.

Supplementary Dataset 9. Results of functional analysis of the FOs from FO db using Inter Pro domain Gene Ontology (GO) slim annotation, including all parent terms. This table provides the Inter Pro domains, manually curated GO annotations for these domains, and direct ancestor GO slim terms to address the differing level of detail in GO annotation between different domains.

Supplementary Dataset 10. DNA and amino acid sequences for all of the coding regions of the plasmids used to express the FOs included in this study. All plasmids have been deposited with Addgene, and both DNA and amino acid sequences are documented there as well.
