# Supplementary material for: Defining the condensate landscape of fusion oncoproteins
Source: Nat Commun. 2023 Sep 28;14:6008. doi: 10.1038/s41467-023-41655-2 (PMC10539325; doi:10.1038/s41467-023-41655-2)
Supplement: Supplementary file 14 — Reporting Summary [file 41467_2023_41655_MOESM14_ESM.pdf]

nature portfolio

Corresponding author(s): Richard Kriwacki, Nidhi Sahni, Jinghui Zhang, Charles Mullighan, M. Madan Babu, and S. Stephen Yi

Last updated by author(s): Aug 22, 2023

Reporting Summary

Nature Portfolio wishes to improve the reproducibility of the work that we publish. This form provides structure for consistency and transparency in reporting. For further information on Nature Portfolio policies, see our Editorial Policies and the Editorial Policy Checklist.

Statistics

For all statistical analyses, confirm that the following items are present in the figure legend, table legend, main text, or Methods section.

n/a	Confirmed	
☐	☒	The exact sample size (*n*) for each experimental group/condition, given as a discrete number and unit of measurement
☒	☐	A statement on whether measurements were taken from distinct samples or whether the same sample was measured repeatedly
☐	☒	The statistical test(s) used AND whether they are one- or two-sided<br>*Only common tests should be described solely by name; describe more complex techniques in the Methods section.*
☒	☐	A description of all covariates tested
☒	☐	A description of any assumptions or corrections, such as tests of normality and adjustment for multiple comparisons
☐	☒	A full description of the statistical parameters including central tendency (e.g. means) or other basic estimates (e.g. regression coefficient) AND variation (e.g. standard deviation) or associated estimates of uncertainty (e.g. confidence intervals)
☐	☒	For null hypothesis testing, the test statistic (e.g. *F*, *t*, *r*) with confidence intervals, effect sizes, degrees of freedom and *P* value noted<br>*Give P values as exact values whenever suitable.*
☒	☐	For Bayesian analysis, information on the choice of priors and Markov chain Monte Carlo settings
☒	☐	For hierarchical and complex designs, identification of the appropriate level for tests and full reporting of outcomes
☐	☒	Estimates of effect sizes (e.g. Cohen's *d*, Pearson's *r*), indicating how they were calculated

Our web collection on statistics for biologists contains articles on many of the points above.

Software and code

Policy information about availability of computer code

Data collection	Slidebook (RRID:SCR_014300) 6.0 (3i) was used to collect all images. The details of softwares used for data collection in this study are also described in the Methods.
Data analysis	All original code has been deposited to Zenodo: 10.5281/zenodo.7114870 and is available at the provided link.<br><br>Data was analyzed as described in the Methods using the following software and tools: ChimerDB 3.0 (ChimerKB, ChimerPub and ChimerSeq), TopHat-Fusion, FusionScan, Ensembl 75, the CICERO gene fusion caller, Transeq, the emboss suite v6.6.0, Fuzzion2, NCBI BLAST, Image J 1.54f, CIDER, the Conserved Domain Database, the SEG algorithm, NCBI's Conserved Domain Database, PLAAC, IUPred2, NLStradamus, PAPA algorithm, infotheo (version: 1.2.0) package, R (version: 4.1.0), rstatix R package (version: 0.7.0), effsize R package (version: 0.8.1), the pheatmap R package (version: 1.0.12), pvclust R package (version 2.2-0), MUSCLE (MUltiple Sequence Comparison by Log-Expectation), PhaSepDB 1.0, DrLLPS 1.0, PhaSePro, LLPSDB, UniProt release 2022_03, eulerr R package, InterProScan 5.52, InterPro 86.0, mapslim function of the GOATOOLS Python library, the Trackmate plugin for Image J, Intersection-over-Union (IoU) overlap tracker in Image J, the h2o.ai (version: 3.36.1.1), catGranule, DeePhase, FuzDrop, R packages PRROC (version 1.3.1) and MLmetrics (version 1.1.1), h2o.predcit_contribution function in the h2o.ai package in R, Hmisc package (version 4.7.1) in R, and Analyze Network option in Cytoscape (version 3.9.1).

For manuscripts utilizing custom algorithms or software that are central to the research but not yet described in published literature, software must be made available to editors and reviewers. We strongly encourage code deposition in a community repository (e.g. GitHub). See the Nature Portfolio guidelines for submitting code & software for further information.

Data

Policy information about availability of data

All manuscripts must include a data availability statement. This statement should provide the following information, where applicable:

- Accession codes, unique identifiers, or web links for publicly available datasets
- A description of any restrictions on data availability
- For clinical datasets or third party data, please ensure that the statement adheres to our policy

All plasmids generated in this study have been deposited to Addgene.For all imaged fusion oncoproteins (FOs) and controls, cellular image files are provided in raw format on the BioImage archive ((https://www.ebi.ac.uk/bioimage-archive/) with the accession number: S-BIAD863.

The data used for sequence analyses are provided in the supplementary datasets. All the raw data has been deposited to Zenodo: 10.5281/zenodo.7114870 and is available at the provided link.

Research involving human participants, their data, or biological material

Policy information about studies with human participants or human data. See also policy information about sex, gender (identity/presentation), and sexual orientation and race, ethnicity and racism.

Reporting on sex and gender	No human research was included in the manuscript.
Reporting on race, ethnicity, or other socially relevant groupings	No human research was included in the manuscript.
Population characteristics	No human research was included in the manuscript.
Recruitment	No human research was included in the manuscript.
Ethics oversight	No human research was included in the manuscript.

Note that full information on the approval of the study protocol must also be provided in the manuscript.

Field-specific reporting

Please select the one below that is the best fit for your research. If you are not sure, read the appropriate sections before making your selection.

☒ Life sciences ☐ Behavioural & social sciences ☐ Ecological, evolutionary & environmental sciences

For a reference copy of the document with all sections, see nature.com/documents/nr-reporting-summary-flat.pdf

Life sciences study design

All studies must disclose on these points even when the disclosure is negative.

Sample size	At least 50 cells were recorded for each FO, based upon 2 biological replicates. No sample size calculation was performed. This number was chosen due to limitations in the throughput of the assay (nearly 200 different fusions were imaged), but the phenotypes seen were consistent and reproducible at this level of replication.
Data exclusions	Untransfected (non-expressing) cells were excluded from analysis. As described in the methods, a cell was considered non-expressing if the minimum mEGFP intensity of the expressing region was less than 50 raw fluorescence intensity units above background. This cut-off was chosen by comparing the minimum mEGFP intensity per pixel (mEGFP-FO expression level) over the puncta(+) FO image datasets.
Replication	All attempts at replication were successful. At least two replicates were performed for imaging of each FO.
Randomization	No randomization was performed because the observation of puncta(+) or puncta(-) behavior by FOs was highly reproducible (including on different days and different locations within 96 well plates).
Blinding	Investigators were not blinded to FO names during puncta(+) or puncta(-) classification as this information was meaningless without sequence feature information, which was only calculated after classification of each FO was performed. Therefore, the assays of puncta formation were unbiased. However, for the 29 Verification FOs used to test the accuracy of the model, authors were blind to the FO-Punca ML model prediction for each FO at the time of imaging and quantification.

Reporting for specific materials, systems and methods

We require information from authors about some types of materials, experimental systems and methods used in many studies. Here, indicate whether each material, system or method listed is relevant to your study. If you are not sure if a list item applies to your research, read the appropriate section before selecting a response.

Materials & experimental systems

n/a	Involved in the study
☒	☐ Antibodies
☐	☒ Eukaryotic cell lines
☒	☐ Palaeontology and archaeology
☒	☐ Animals and other organisms
☒	☐ Clinical data
☒	☐ Dual use research of concern
☒	☐ Plants

Methods

n/a	Involved in the study
☒	☐ ChIP-seq
☒	☐ Flow cytometry
☒	☐ MRI-based neuroimaging

Eukaryotic cell lines

Policy information about cell lines and Sex and Gender in Research

Cell line source(s)	HeLa cells were obtained from ATCC (RRID:CVCL_0030).
Authentication	Cells were authenticated by STR-profiling using PowerPlex Fusion at the St. Jude Hartwell Center.
Mycoplasma contamination	Cells were tested for Mycoplasma every 2 months using PCR (e-MycoTM plus, LiLiF) and a negative result was obtained each time.
Commonly misidentified lines (See ICLAC register)	No commonly misidentified cell lines were used in the study.
